# Coffee consumption and overall and cause-specific mortality: the Norwegian Women and Cancer Study (NOWAC)

**DOI:** 10.1007/s10654-020-00664-x

**Published:** 2020-07-23

**Authors:** Marko Lukic, Runa Borgund Barnung, Guri Skeie, Karina Standahl Olsen, Tonje Braaten

**Affiliations:** 1 Department of Community Medicine, Faculty of Health Sciences, UiT The Arctic University of Norway, Tromsø, Norway; 2 Institutt for Samfunnsmedisin, UiT Norges Arktiske Universitet, 9037 Tromsø, Norway

**Keywords:** Coffee, Mortality, Cancer mortality, Cardiovascular mortality, Prospective cohort study

## Abstract

Coffee consumption has previously been reported to reduce overall and cause-specific mortality. We aimed to further investigate this association by coffee brewing methods and in a population with heavy coffee consumers. The information on total, filtered, instant, and boiled coffee consumption from self-administered questionnaires was available from 117,228 women in the Norwegian Women and Cancer (NOWAC) Study. We used flexible parametric survival models to calculate hazard ratios (HR) and 95% confidence intervals (CI) for all-cause, cardiovascular, and cancer mortality by total coffee consumption and brewing methods, and adjusted for smoking status, number of pack-years, age at smoking initiation, alcohol consumption, body mass index, physical activity, and duration of education. During 3.2 million person-years of follow-up, a total of 16,106 deaths occurred. Compared to light coffee consumers (≤ 1 cup/day), we found a statistically significant inverse association with high-moderate total coffee consumption (more than 4 and up to 6 cups/day, HR 0.89; 95% CI 0.83–0.94) and all-cause mortality. The adverse association between heavy filtered coffee consumption (> 6 cups/day) and all-cause mortality observed in the entire sample (HR 1.09; 95% CI 1.01–1.17) was not found in never smokers (HR 0.85; 95% CI 0.70–1.05). During the follow-up, both high-moderate total and filtered coffee consumption were inversely associated with the risk of cardiovascular mortality (HR 0.79; 95% CI 0.67–0.94; HR 0.80; 95% CI 0.67–0.94, respectively). The association was stronger in the analyses of never smokers (> 6 cups of filtered coffee/day HR 0.20; 95% CI 0.08–0.56). The consumption of more than 6 cups/day of filtered, instant, and coffee overall was found to increase the risk of cancer deaths during the follow-up. However, these associations were not statistically significant in the subgroup analyses of never smokers. The data from the NOWAC study indicate that the consumption of filtered coffee reduces the risk of cardiovascular deaths. The observed adverse association between coffee consumption and cancer mortality is most likely due to residual confounding by smoking.

**Electronic supplementary material:**

The online version of this article (10.1007/s10654-020-00664-x) contains supplementary material, which is available to authorized users.

## Introduction

Average consumption of coffee in Norway reached almost 10 kg per capita during the previous decade [1]. Filtered coffee continues to be the most popular brewing method, followed by boiled and instant coffee as the alternative brewing methods [2]. According to a survey from 2017, 70% of Norwegians are drinking coffee daily [3].

Coffee may influence risk of disease through molecular and physiological mechanisms. Coffee and its constituents can increase carcinogen detoxification [4], affect DNA methylation [5], increase insulin sensitivity [6], positively impact liver health [7], and in addition, immunological mechanisms have been reported [5, 8]. Still, overall effects on lipid metabolism remains to be established [9]. Finally, caffeine, chlorogenic acid, kahweol, cafestol, and coffee maillard reaction products are all found to have anti-oxidant properties [10–14]. Via these numerous mechanisms, coffee and its constituents may influence human health, and thereby potentially affect the risk of mortality.

Concentration and bioavailability of the constituents found in coffee depend on brewing methods. Coffee diterpens (kahweol and cafestol), as well as caffeine are found in higher concentrations in boiled compared to filtered coffee [15, 16], similarly, the anti-oxidant activity of chlorogenic acid is found to be higher in boiled compared to other coffee brews [15].

In a recent umbrella review on coffee, caffeine and health outcomes [14], coffee was found to be associated with a probable decreased risk of some cancer types (liver, endometrial), CVD and CVD mortality, Parkinson’s disease, and type 2 diabetes. Caffeine was found to probably decrease the risk of Parkinson’s and type 2 diabetes, but increased the risk of pregnancy loss.

To this day, there are several meta-analyses conducted in order to quantify the risk of overall and cause specific mortality in regards to coffee consumption [17–20]. The results from the meta-analyses all indicated that coffee consumption was associated with a lower risk of all-cause mortality, and in two meta-analyses, coffee consumption reduced the risk of mortality from cardiovascular diseases (CVD). The association of coffee consumption with reduced cancer mortality was not as clear as for all-cause and CVD mortality [17]. However, data is lacking on the potential effects of different coffee brewing types on these outcomes.

In 2017, ischemic heart diseases were the leading causes of deaths in Norway, followed by Alzheimer’s disease, stroke, chronic obstructive pulmonary disease, and lung cancer [21]. In the present study, we aimed to investigate the association between high coffee consumption by different brewing methods and all-cause, CVD, and cancer mortality.

## Materials and methods

### Study sample

The Norwegian Women and Cancer (NOWAC) study is a nationally representative, population based cohort study that includes approximately 172,000 women. The NOWAC study has been described in detail elsewhere [22]. In short, the study was initiated in 1991, and random samples of Norwegian women aged 30–70 years was drawn from the Norwegian Central Population Registry and were invited to participate. Our initial study cohort consisted of those women who completed a version of the NOWAC food frequency questionnaire (FFQ) that included questions on coffee consumption by brewing method (filtered, instant, boiled). We excluded women without information on all three brewing methods (N = 55,220). Following these exclusions, our final study sample consisted of 117,228 women.

The NOWAC Study was approved by the Regional Committee for Medical Research Ethics and the Norwegian Data Inspectorate. All women gave written informed consent.

### Assessment of coffee consumption

Women enrolled in the NOWAC study answered the following question on coffee consumption: “How many cups of each kind of coffee (boiled, filtered, instant) did you usually drink during the past year?” For each brewing method, women could choose between the following answers depending on the version of the FFQ: never/seldom, 1–6 cups/week, 1 cup/day, 2–3 cups/day, 4–5 cups/day, 6–7 cups/day, and ≥ 8 cups/day, or never/seldom, 1–3 cups/month, 1 cup/week, 2–4 cups/week, 5–6 cups/week, 1 cup/day, 2–3 cups/day, 4–5 cups/day, and 6–10 cups/day. We assigned a midpoint value for each of the categories, which were then summed up in order to calculate consumption of filtered, boiled, and instant coffee. Total coffee consumption was calculated as the combined consumption of all brewing methods. For total coffee consumption, and within each brewing type, women were categorized as light consumers (≤ 1 cup/day), low-moderate consumers (> 1–4 cups/day), high moderate consumers (> 4–6 cups/day), and heavy consumers (> 6 cups/day).

### End points

We used the unique 11-digit personal number assigned to every legal resident in Norway to obtain information on death and emigration through linkage to the Norwegian Central Population Register. We acquired information on causes of death in our cohort from the National Registry for Causes of Death. We used the 10th Revision of the International Statistical Classification of Diseases, Injuries and Causes of Death (ICD-10) to classify causes into CVD deaths that included stroke, coronary heart disease, and other vascular causes (ICD-10: I00-I99), and cancer deaths (ICD-10: C00-D48). All-cause mortality was defined as the combination of CVD, cancer, and deaths from all other causes.

### Statistical analysis

For the all-cause mortality, person-years were calculated from the beginning of the study follow-up until the date of emigration, death or end of the study period (December 31st 2018), whichever occurred first. For cancer and cardiovascular mortality the end of the study period was set to 31. December 2016, as we had information on causes of death only until the end of 2016.

We used flexible parametric models (by using *stpm2* module [23] in STATA version 15.0, StataCorp, Lakeway Drive, College Station, TX) to obtain hazard ratios (HR) with 95% confidence intervals (CI) for the association between coffee consumption and the risk of CVD, cancer, and all-cause mortality. Light coffee consumers were used as the reference group, as it was not possible to differentiate between coffee abstainers and occasional coffee drinkers because of the answers offered in the FFQ. Attained age was used as the underlying time scale.

The final models for each outcome were adjusted for the following a priori selected covariates: smoking status (current, former, never), age at smoking initiation (< 20, ≥ 20 years), number of pack-years (≤ 14, 15–19, ≥ 20), body mass index (BMI ≤ 18.49, 18.5–24.9, 25–29.9, and ≥ 30 kg/m^2^), alcohol consumption (0, 0.1–3.99, 4–9.99, ≥ 10 g/day), duration of education (≤ 9, 10–12, 13–16, ≥ 17 years), and level of physical activity (self-reported on an increment scale from 1 to 10 and categorized into 1–4, 5–6, 7–10). In the analyses that required adjusting for smoking exposure, we modelled these by combining the information on smoking status, age at smoking initiation, and number of pack-years into five categorical variables. In addition, brewing method-specific analyses were adjusted for the two other brewing methods.

We modeled restricted cubic splines with four knots, with its locations based on Harrell’s recommended percentiles of the total coffee consumption to assess the shape of the relationship between overall coffee consumption and CVD, cancer, and all-cause mortality [24]. A Wald-type test was used to assess if the coefficients of the second and third spline were equal to zero. We calculated a per cup change in risk of the study outcomes by using a generalized least squares for trend estimation of summarized dose–response data [25].

In order to counteract potential residual confounding due to smoking, we conducted subgroup analyses on never smokers for all of the outcomes. Furthermore, we did the analyses in which we had excluded deaths that occurred during the first year of follow-up. We also checked for potential effect modification of BMI, by including an interaction term between BMI and total coffee intake.

## Results

During 3.2 million person-years of follow-up, a total of 16,106 deaths occurred. The median follow-up time was 20.5 years. Most of the study participants reported drinking not more than 4 cups of coffee per day, with filtered coffee being the most frequently consumed (Table 1). The distribution of deaths according to levels of coffee consumption is presented in supplementary Table 1.

**Table 1:** Distribution of participants according to total, filtered, instant, and boiled coffee consumption, the Norwegian Women and Cancer Study, 1991–2016

Light consumers ≤ 1 cup/day	Low moderate consumers > 1–4 cups/day	High moderate consumers > 4–6 cups/day	Heavy consumers > 6 cups/day
*Total coffee consumption*			
25,115 (21.4)	36,400 (31.1)	33,883 (28.9)	21,830 (18.6)
*Filtered coffee consumption*			
≤ 1 cup/day	> 1–4 cups/day	> 4–6 cups/day	> 6 cups/day
51,611 (44.0)	28,431 (24.3)	24,194 (20.6)	12,992 (11.1)
*Instant coffee consumption*			
≤ 1 cup/day	> 1–4 cups/day	> 4–6 cups/day	> 6 cups/day
108,162 (92.3)	5923 (5.1)	2214 (1.9)	929 (0.8)
*Boiled coffee consumption*			
≤ 1 cup/day	> 1–4 cups/day	> 4–6 cups/day	> 6 cups/day
95,254 (81.3)	8726 (7.4)	7823 (6.7)	5425 (4.6)

Compared to heavy coffee consumers, light coffee consumers were younger at the baseline, had more years of attained education, and were more likely to be never smokers. Compared to other levels of coffee consumption, women that drank more than 6 cups of coffee per day were more likely to be current smokers (58%) and were also the heaviest smokers (Table 2).

**Table 2:** Characteristics of the study sample by total coffee consumption, the Norwegian Women and Cancer Study, 1996–2016

Characteristics	Total coffee consumption			
	Light consumers ≤ 1 cup/day	Low moderate consumers More than 1 up to 4 cups/day	High moderate consumers More than 4 up to 6 cups/day	Heavy consumers > 6 cups/day
Participants at baseline, N (%)	25 115 (21.4)	36 400 (31.1)	33 883 (28.9)	21 830 (18.6)
Age at baseline (y), mean (SD)	45.7 (9)	49.2 (8.7)	47.7 (8.4)	46.7 (8.0)
*Smoking status at baseline, %*				
Never	45.9	42.5	30.5	15.9
Former	30.4	35.9	32.9	26.1
Current	23.7	21.6	36.6	58.0
Age at smoking initiation (y), mean (SD)	19.1 (4.8)	19.6 (5.5)	19.7 (5.4)	19.6 (5.6)
Number of pack-years smoked at baseline, mean (SD)	7.6 (7.6)	7.7 (8.1)	9.2 (8.0)	12.4 (8.8)
Duration of education (y), mean (SD)	13.1 (3.6)	12.5 (3.5)	11.7 (3.3)	10.9 (3.1)
Body mass index, mean (SD)	23.9 (4.1)	24.0 (3.8)	24.0 (3.7)	24.1 (3.9)
Physical activity level, mean (SD)	5.5 (1.9)	5.7 (1.8)	5.6 (1.9)	5.5 (2.1)
Alcohol consumption (g/day), mean (SD)	3.1 (6.2)	3.4 (4.9)	3.2 (4.6)	3.2 (7.0)

*SD* standard deviation

**Fig. 1:**
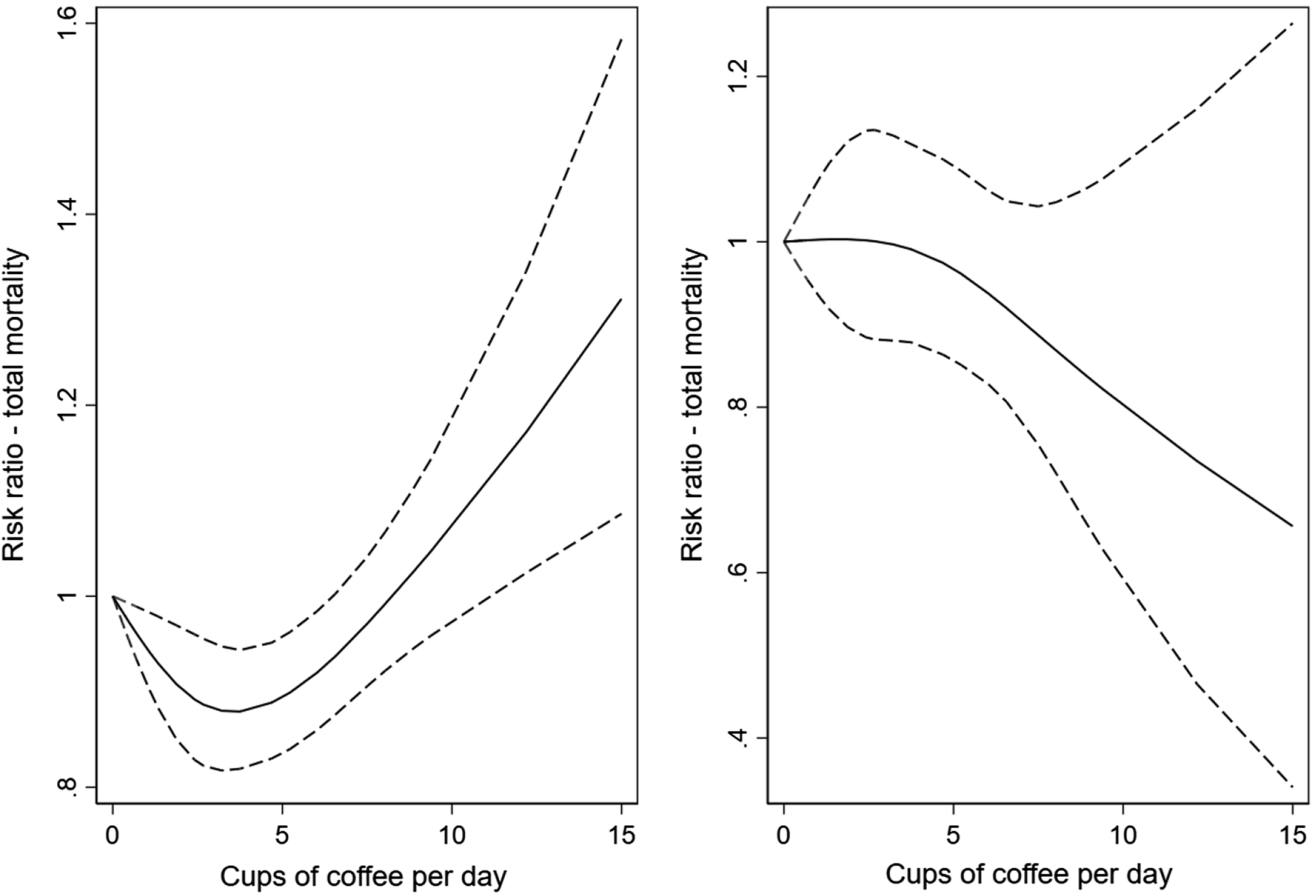
Spline regression models for total coffee intake in relation to all-cause mortality in a whole sample (**a**) and in never-smokers (**b**). (Solid lines—HR, dashed lines—95% CE)

**Fig. 2:**
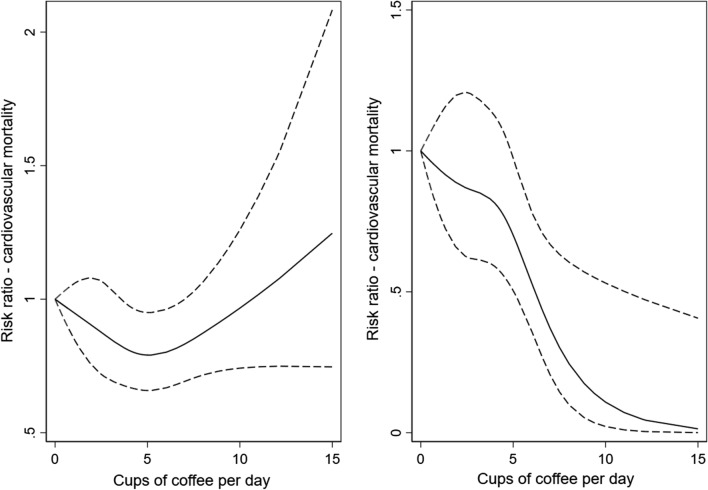
Spline regression models for total coffee intake in relation to cardiovascular mortality in a whole sample (**a**) and in never-smokers (**b**). (Solid lines—HR, dashed lines—95% CE)

**Fig. 3:**
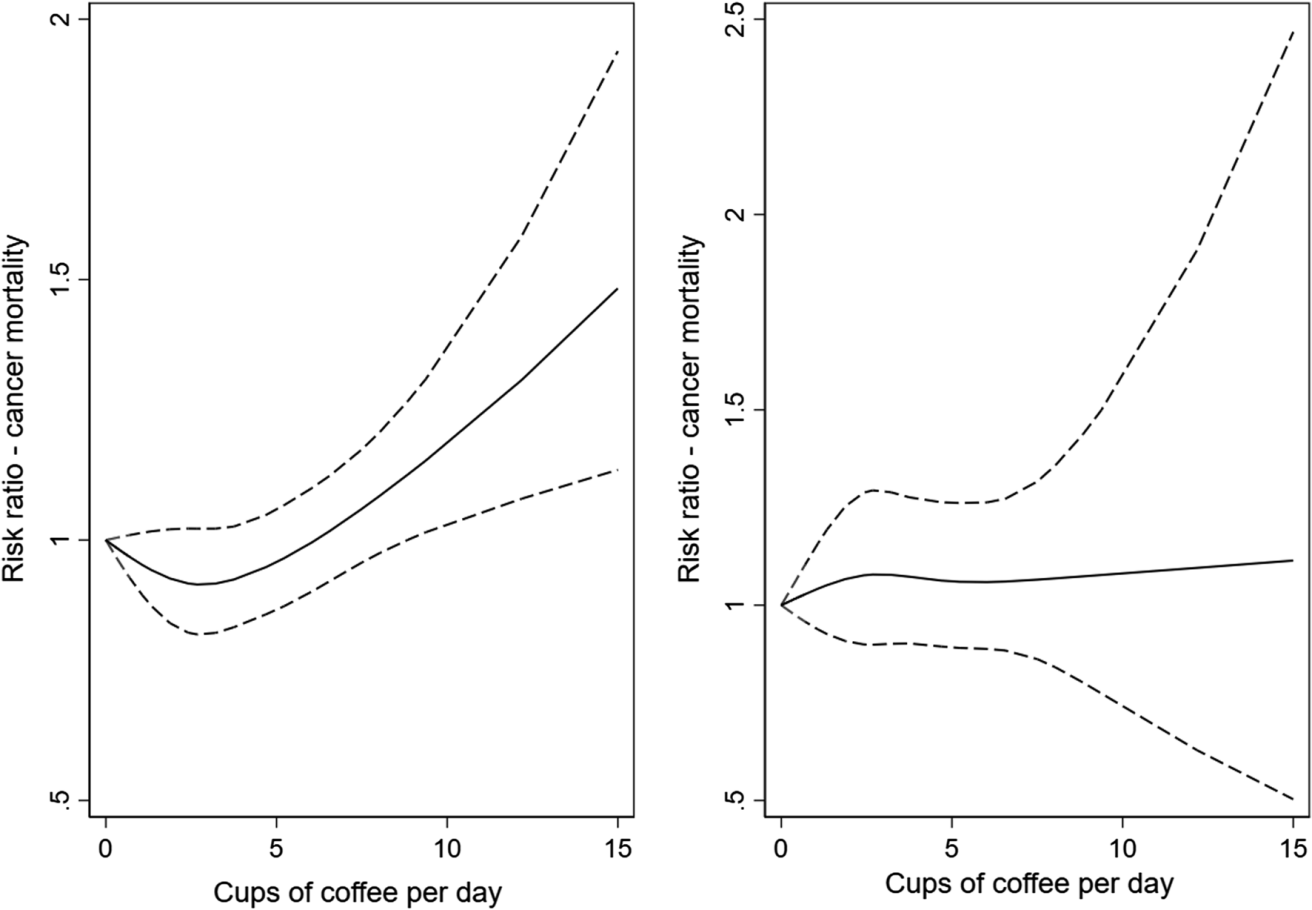
Spline regression models for total coffee intake in relation to cancer mortality in a whole sample (**a**) and in never-smokers (**b**). (Solid lines—HR, dashed lines—95% CE)

Compared to light coffee consumers, high-moderate coffee consumers (> 4–6 cups/day) had a lower risk of deaths from all causes (HR 0.89, 95% CI 0.83–0.94), and of CVD mortality (HR 0.79, 95% CI 0.67–0.94, Tables 3 and 5, Fig. 1) during the follow-up. In the analyses of never smokers, we found a strong inverse association between heavy coffee drinking and CVD mortality (HR 0.32, 95% CI 0.17–0.60), and observed a strong dose–response relationship with one additional cup of coffee per day being associated with a 9% lower risk of CVD deaths (95% CI 4–14%, Table 6, Fig. 2). During the follow-up, a slightly increased risk of cancer deaths was observed in heavy coffee consumers compared to the reference group (HR 1.14, 95% CI 1.03–1.26, Table 7, Fig. 3). However, no statistically significant association with cancer deaths were found in never smokers (HR 1.09, 95% CI 0.88–1.36, Table 8, Fig. 3).

**Table 3:** Hazard ratios (HRs) with 95% confidence intervals (CI) of all-cause mortality according to total, filtered, instant, and boiled coffee consumption in the Norwegian Women and Cancer Study

All-cause mortality								
Coffee consumption	Total coffee consumption		Filtered coffee consumption		Instant coffee consumption		Boiled coffee consumption	
	Age-adjusted N = 117 228 n = 12 364	Multivariable^a^ N = 98 553 n = 9309	Age-adjusted N = 117,228 n = 12 364	Multivariable^b^ N = 98 553 n = 9309	Age-adjusted N = 117,228 n = 12 364	Multivariable^b^ N = 98 553 n = 9309	Age-adjusted N = 117,228 n = 12 364	Multivariable^b^ N = 98 553 n = 9309
	HR 95% CI	HR 95% CI	HR 95% CI	HR 95% CI	HR 95% CI	HR 95% CI	HR 95% CI	HR 95% CI
≤ 1 cup/day	1.00	1.00	1.00	1.00	1.00	1.00	1.00	1.00
> 1–4 cups/day	0.88 (0.83–0.93)	0.90 (0.84–0.95)	0.87 (0.82–0.91)	0.91 (0.85–0.96)	0.89 (0.82–0.96)	0.91 (0.83–1.00)	1.06 (0.99–1.14)	0.99 (0.91–1.07)
> 4–6 cups/day	1.00 (0.95–1.06)	0.89 (0.83–0.94)	1.00 (0.95–1.06)	0.90 (0.85–0.96)	1.13 (0.99–1.28)	0.97 (0.85–1.11)	1.16 (1.01–1.28)	0.96 (0.99–1.05)
> 6 cups/day	1.43 (1.36–1.52)	1.02 (0.95–1.09)	1.50 (1.42–1.59)	1.09 (1.01–1.17)	1.63 (1.39–1.92)	1.11 (0.93–1.34)	1.47 (1.37–1.59)	0.98 (0.89–1.08)
Per cup increase	1.00 (0.99–1.01)		1.00 (0.99–1.01)		1.00 (0.98–1.01)		0.99 (0.98–0.99)	

*Cat.* categorical

^a^Adjusted for smoking status, age at smoking initiation, number of pack-years smoked, body mass index (cat.), alcohol consumption (g/day) (cat.), physical activity (cat.), years of attained education (cat.)

^b^Adjusted for smoking status, age at smoking initiation, number of pack-years smoked, body mass index (cat.), alcohol consumption (g/day) (cat.), physical activity (cat.), years of attained education (cat.), and mutually adjusted for the consumption of coffee brewed with two other methods (cat.)

Finally, there was a significant departure from linearity as shown by the Wald-type test in the association between total coffee consumption and the total, CVD, and cancer mortality in the whole sample (*p* < 0.001, *p* = 0.03, *p* = 0.005, respectively). However, no significant departure was found in never smokers (*p* = 0.62, *p* = 0.17, *p* = 0.79, respectively).

For filtered coffee, consuming more than 6 cups per day was associated with a slightly increased risk of all-cause mortality in the whole sample (HR 1.09, 95% CI 1.01–1.17), but not in never smokers (HR 0.85, 95% CI 0.70–1.05, Tables 3 and 4). A low-moderate and high-moderate consumption of filtered coffee was associated with the reduced risk of CVD mortality (Table 5). In the analyses of never-smokers, we found an even stronger association, with a strong inverse association between heavy filtered coffee consumption and CVD deaths (HR 0.20; 95% CI 0.08–0.56, Table 6). At the same time, one additional cup of filtered coffee per day was associated with a 10% lower risk of CVD deaths (95% CI 5–16%, Fig. 2). We found a 23% increased risk of cancer mortality among heavy filtered coffee consumers (95% CI 11–36%, Table 7), but no significant associations between filtered coffee consumption and cancer deaths were found in never-smokers (Table 8).

**Table 4:** Hazard ratios (HRs) with 95% confidence intervals (CI) of all-cause mortality according to total, filtered, instant, and boiled coffee consumption in the Norwegian Women and Cancer Study-never smokers

All-cause mortality								
Coffee consumption	Total coffee consumption		Filtered coffee consumption		Instant coffee consumption		Boiled coffee consumption	
	Age-adjusted N = 40,232 n = 3319	Multivariable^a^ N = 34,360 n = 2511	Age-adjusted N = 40,232 n = 3319	Multivariable^b^ N = 34,360 n = 2511	Age-adjusted N = 40,232 n = 3319	Multivariable^b^ N = 34,360 n = 2511	Age-adjusted N = 40,232 n = 3319	Multivariable^b^ N = 34,360 n = 2511
	HR 95% CI	HR 95% CI	HR 95% CI	HR 95% CI	HR 95% CI	HR 95% CI	HR 95% CI	HR 95% CI
≤ 1 cup/day	1.00	1.00	1.00	1.00	1.00	1.00	1.00	1.00
> 1–4 cups/day	0.92 (0.85–1.01)	0.96 (0.87–1.07)	0.87 (0.80–0.95)	0.96 (0.87–1.06)	0.99 (0.85–1.14)	1.01 (0.85–1.19)	1.09 (0.98–1.22)	1.05 (0.92–1.20)
> 4–6 cups/day	0.91 (0.83–1.01)	0.94 (0.84–1.05)	0.88 (0.80–0.98)	0.92 (0.82–1.04)	1.09 (0.86–1.40)	1.11 (0.84–1.46)	0.97 (0.84–1.12)	0.93 (0.78–1.11)
> 6 cups/day	0.91 (0.79–1.04)	0.88 (0.75–1.03)	0.90 (0.76–1.06)	0.85 (0.70–1.05)	1.2 (0.76–1.97)	1.17 (0.69–1.99)	0.85 (0.66–1.09)	0.80 (0.58–1.09)
Per cup increase	0.98 (0.97–1.00)		0.98 (0.96–0.99)		1.02 (0.98–1.06)		0.99 (0.96–1.01)	

*Cat.* categorical

^a^Adjusted for body mass index (cat.), alcohol consumption (g/day) (cat.), physical activity (cat.), years of attained education (cat.)

^b^Adjusted for body mass index (cat.), alcohol consumption (g/day) (cat.), physical activity (cat.), years of attained education (cat.), and mutually adjusted for the consumption of coffee brewed with two other methods (cat.)

**Table 5:** Hazard ratios (HRs) with 95% confidence intervals (CI) of cardiovascular mortality according to total, filtered, instant, and boiled coffee consumption in the Norwegian Women and Cancer Study

Cardiovascular mortality								
Coffee consumption	Total coffee consumption		Filtered coffee consumption		Instant coffee consumption		Boiled coffee consumption	
	Age-adjusted N = 117 228 n = 1953	Multivariable^a^ N = 98 553 n = 1328	Age-adjusted N = 117 228 n = 1953	Multivariable^b^ N = 98 553 n = 1328	Age-adjusted N = 117 228 n = 1953	Multivariable^b^ N = 98 553 n = 1328	Age-adjusted N = 117 228 n = 1953	Multivariable^b^ N = 98 553 n = 1328
	HR 95% CI	HR 95% CI	HR 95% CI	HR 95% CI	HR 95% CI	HR 95% CI	HR 95% CI	HR 95% CI
≤ 1 cup/day	1.00	1.00	1.00	1.00	1.00	1.00	1.00	1.00
> 1–4 cups/day	0.80 (0.70–0.92)	0.85 (0.72–1.00)	0.76 (0.66–0.86)	0.80 (0.68–0.94)	0.80 (0.64–0.99)	0.94 (0.74–1.20)	1.01 (0.86–1.19)	0.93 (0.76–1.15)
> 4–6 cups/day	0.92 (0.81–1.06)	0.79 (0.67–0.94)	0.91 (0.79–1.04)	0.80 (0.67–0.94)	1.11 (0.83–1.47)	0.95 (0.68–1.32)	1.10 (0.92–1.30)	0.89 (0.72–1.10)
> 6 cups/day	1.32 (1.15–1.52)	0.85 (0.71–1.02)	1.43 (1.22–1.66)	0.92 (0.76–1.12)	0.96 (0.60–1.53)	0.74 (0.43–1.27)	1.54 (1.28–1.85)	0.90 (0.70–1.15)
Per cup increase	0.98 (0.96–1.00)		0.98 (0.96–1.00)		0.97 (0.93–1.02)		0.98 (0.95–1.01)	

*Cat.* categorical

^a^Adjusted for smoking status, age at smoking initiation, number of pack-years smoked, body mass index (cat.), alcohol consumption (g/day) (cat.), physical activity (cat.), years of attained education (cat.)

^b^Adjusted for smoking status, age at smoking initiation, number of pack-years smoked, body mass index (cat.), alcohol consumption (g/day) (cat.), physical activity (cat.), years of attained education (cat.), and mutually adjusted for the consumption of coffee brewed with two other methods (cat.)

**Table 6:** Hazard ratios (HRs) with 95% confidence intervals (CI) of cardiovascular mortality according to total, filtered, instant, and boiled coffee consumption in the Norwegian Women and Cancer Study—never smokers

Cardiovascular mortality								
Coffee consumption	Total coffee consumption		Filtered coffee consumption		Instant coffee consumption		Boiled coffee consumption	
	Age-adjusted N = 40 232 n = 505	Multivariable^a^ N = 34 360 n = 345	Age-adjusted N = 40 232 n = 505	Multivariable^b^ N = 34 360 n = 345	Age-adjusted N = 40 232 n = 505	Multivariable^b^ N = 34 360 n = 345	Age-adjusted N = 40 232 n = 505	Multivariable^b^ N = 34 360 n = 345
	HR 95% CI	HR 95% CI	HR 95% CI	HR 95% CI	HR 95% CI	HR 95% CI	HR 95% CI	HR 95% CI
≤ 1 cup/day	1.00	1.00	1.00	1.00	1.00	1.00	1.00	1.00
> 1–4 cups/day	0.81 (0.65–1.00)	0.85 (0.65–1.10)	0.70 (0.55–0.87)	0.75 (0.57–0.98)	0.66 (0.44–0.98)	0.71 (0.44–1.13)	0.91 (0.69–1.21)	0.86 (0.61–1.23)
> 4–6 cups/day	0.74 (0.58–0.95)	0.75 (0.56–1.02)	0.67 (0.51–0.89)	0.71 (0.51–0.98)	0.77 (0.39–1.50)	0.90 (0.44–1.85)	0.97 (0.39–1.36)	0.94 (0.61–1.43)
> 6 cups/day	0.43 (0.28–0.68)	0.32 (0.17–0.60)	0.39 (0.21–0.71)	0.20 (0.08–0.56)	0.79 (0.20–3.18)	0.95 (0.23–3.83)	0.42 (0.18–0.94)	0.32 (0.10–1.02)
Per cup increase	0.91 (0.86–0.96)		0.90 (0.84–0.95)		0.95 (0.86–1.05)		0.95 (0.88–1.02)	

*Cat.* categorical

^a^Adjusted for body mass index (cat.), alcohol consumption (g/day) (cat.), physical activity (cat.), years of attained education (cat.)

^b^Adjusted for body mass index (cat.), alcohol consumption (g/day) (cat.), physical activity (cat.), years of attained education (cat.), and mutually adjusted for the consumption of coffee brewed with two other methods (cat.)

**Table 7:** Hazard ratios (HRs) with 95% confidence intervals (CI) of cancer mortality according to total, filtered, instant, and boiled coffee consumption in the Norwegian Women and Cancer Study

Cancer mortality								
Coffee consumption	Total coffee consumption		Filtered coffee consumption		Instant coffee consumption		Boiled coffee consumption	
	Age-adjusted N = 117 228 n = 5483	Multivariable^a^ N = 98 553 n = 4344	Age-adjusted N = 117 228 n = 5483	Multivariable^b^ N = 98 553 n = 4344	Age-adjusted N = 117 228 n = 5483	Multivariable^b^ N = 98 553 n = 4344	Age-adjusted N = 117 228 n = 5483	Multivariable^b^ N = 98 553 n = 4344
	HR 95% CI	HR 95% CI	HR 95% CI	HR 95% CI	HR 95% CI	HR 95% CI	HR 95% CI	HR 95% CI
≤ 1 cup/day	1.00	1.00	1.00	1.00	1.00	1.00	1.00	1.00
> 1–4 cups/day	0.96 (0.89–1.05)	0.95 (0.86–1.04)	0.95 (0.89–1.03)	0.97 (0.89–1.05)	0.93 (0.82–1.06)	0.96 (0.83–1.10)	1.09 (0.98–1.21)	1.00 (0.89–1.13)
> 4–6 cups/day	1.07 (0.98–1.16)	0.94 (0.86–1.04)	1.08 (0.99–1.17)	0.97 (0.88–1.06)	1.01 (0.84–1.22)	0.87 (0.70–1.08)	1.21 (1.09–1.34)	1.07 (0.94–1.20)
> 6 cups/day	1.50 (1.38–1.63)	1.14 (1.03–1.26)	1.59 (1.45–1.73)	1.23 (1.11–1.36)	1.71 (1.37–2.15)	1.40 (1.09–1.81)	1.37 (1.22–1.54)	0.98 (0.85–1.13)
Per cup increase	1.02 (1.01–1.03)		1.02 (1.01–1.03)		1.01 (0.99–1.04)		1.00 (0.99–1.02)	

Cat.: categorical

^a^Adjusted for smoking status, age at smoking initiation, number of pack-years smoked, body mass index (cat.), alcohol consumption (g/day) (cat.), physical activity (cat.), years of attained education (cat.)

^b^Adjusted for smoking status, age at smoking initiation, number of pack-years smoked, body mass index (cat.), alcohol consumption (g/day) (cat.), physical activity (cat.), years of attained education (cat.), and mutually adjusted for the consumption of coffee brewed with two other methods (cat.)

**Table 8:** Hazard ratios (HRs) with 95% confidence intervals (CI) of cancer mortality according to total, filtered, instant, and boiled coffee consumption in the Norwegian Women and Cancer Study—never smokers

Cancer mortality								
Coffee consumption	Total coffee consumption		Filtered coffee consumption		Instant coffee consumption		Boiled coffee consumption	
	Age-adjusted N = 40,232 n = 1513	Multivariable^a^ N = 34,360 n = 1226	Age-adjusted N = 40,232 n = 1513	Multivariable^b^ N = 34,360 n = 1226	Age-adjusted N = 40,232 n = 1513	Multivariable^b^ N = 34,360 n = 1226	Age-adjusted N = 40,232 n = 1513	Multivariable^b^ N = 34,360 n = 1226
	HR 95% CI	HR 95% CI	HR 95% CI	HR 95% CI	HR 95% CI	HR 95% CI	HR 95% CI	HR 95% CI
≤ 1 cup/day	1.00	1.00	1.00	1.00	1.00	1.00	1.00	1.00
> 1–4 cups/day	1.02 (0.90–1.17)	1.05 (0.91–1.22)	0.94 (0.83–1.07)	1.02 (0.88–1.17)	0.97 (0.78–1.21)	1.00 (0.78–1.28)	1.12 (0.95–1.33)	1.00 (0.78–1.28)
> 4–6 cups/day	1.00 (0.87–1.15)	1.01 (0.86–1.19)	1.03 (0.89–1.19)	1.07 (0.91–1.25)	0.97 (0.66–1.44)	0.84 (0.52–1.34)	0.91 (0.72–1.14)	0.84 (0.52–1.34)
> 6 cups/day	1.10 (0.91–1.34)	1.09 (0.88–1.36)	1.07 (0.85–1.35)	1.06 (0.82–1.38)	0.96 (0.43–2.16)	0.91 (0.38–2.21)	1.04 (0.73–1.46)	0.91 (0.38–2.21)
Per cup increase	1.01 (0.98–1.03)		1.01 (0.99–1.04)		0.98 (0.93–1.05)		0.99 (0.96–1.03)	

*Cat.* categorical

^a^Adjusted for body mass index (cat.), alcohol consumption (g/day) (cat.), physical activity (cat.), years of attained education (cat.)

^b^Adjusted for body mass index (cat.), alcohol consumption (g/day) (cat.), physical activity (cat.), years of attained education (cat.), and mutually adjusted for the consumption of coffee brewed with two other methods (cat.)

We did not observe an association between instant coffee consumption and all-cause and CVD mortality during the follow-up (Tables 3 and 5). Heavy instant coffee consumption was associated with a risk of cancer deaths in the whole sample (HR 1.40, 95% CI 1.09–1.81, Table 7), but not in never-smokers (HR 0.91, 95% CI 0.38–2.21, Table 8).

No association was found between boiled coffee consumption and any of the outcomes. We did, however observed a borderline non-significant lower risk of CVD deaths among never smokers who drank more than 6 cups of boiled coffee per day compared to the reference group (HR 0.32, 95% CI 0.10–1.02, Table 6). The results from the analyses with and without smoking adjustment are presented in the supplementary Tables 2–4.

The risk estimates from the lag analyses were similar to those from the analyses that included the entire study sample (results not shown). We did not find evidence that BMI was an effect modifier in the association between total coffee consumption and any of the studied outcomes (results not shown).

## Discussion

The present study used data from the NOWAC study, a large prospective population based cohort of females, to investigate a possible association between coffee consumption, different coffee brewing methods, and all-cause, CVD, and cancer mortality. We found that, at large, total and filtered coffee consumption was associated with reduced all-cause and CVD mortality. However, in the full cohort, the highest coffee consumption (more than 6 cups/day) was associated with increased mortality, but this phenomenon is likely due to residual confounding by smoking, as the association is not present in the non-smokers. Similarly, the association between coffee and increased cancer mortality in the full cohort is not present in the non-smoking sub-group analysis.

A recent umbrella review concluded that coffee may be viewed as part of a healthy diet [14]. This is in line with our results on reduced all-cause and CVD mortality, particularly in non-smokers. In some subgroups of our results (according to outcome, brewing type, smoking status), we find tendency towards lower mortality by dose of coffee, as seen in the non-smokers for CVD mortality. As summarized in the umbrella review, beneficial effects of coffee was found for a number of cancer outcomes, metabolism-related outcomes including CVD, as well as neurological conditions. More specifically, for all-cause mortality, beneficial effects of coffee has been identified in both European [26–28] and American [29–31] cohorts, as well as in three meta-analyses [17, 32, 33]. The same overall picture is true for three meta-analyses [17, 32, 33], and several single cohorts studying CVD mortality [26, 28, 29]. The potential dose–response relationship between coffee and CVD mortality in our results is also recognizable from the majority of the available data: Grosso discusses that there may be a linear dose/response relationship, alternatively that 4–5 cups/day is the most beneficial dose [14].

We found an increased risk of cancer mortality when consuming more than 6 cups of coffee per day (total, filtered, and instant), but this was not significant when looking at the non-smokers. This suggest that residual confounding from smoking still influences the multivariate analysis of the whole cohort. Several meta-analyses have previously considered the association between coffee and cancer mortality. Kim et al. found a non-linear inverse association between coffee consumption and cancer mortality [33]. However, the association between coffee and cancer mortality was not apparent at higher coffee consumptions, and the authors suggest that this might be due to residual confounding from smoking. Similarly, a meta-analysis by Grosso et al. only found a risk reduction for cancer mortality when looking at never smokers [32]. However, no association was found in a third meta-analysis [17]. The residual smoking confounding is also likely seen in the pooled analysis of Ding et al., who identified an increased risk for lung cancer deaths by coffee consumption, but not in sub-group analysis of never smokers [31]. Indeed, when looking at the never smokers, they found an inverse association between coffee consumption (3.1–5 cups/day) and cancer mortality.

### Mechanisms

Several hypotheses have been put forward to explain the association between coffee and health, related to the bioactive compounds found in coffee. However, the concentration of these compounds vary according to coffee species, genetics, roasting and brewing types [34]. In addition, heterogeneity in issues related to study design (doses, duration, choice of biomarker, measurement methods) adds to the variability of reported findings on bioactive constituents [35]. In line with this, we do see some differences in the results according to brewing type, with boiled coffee being the least favorable.

Compounds like diterpenes, trigonelline, melanoidins, caffeine, and chlorogenic acid all have known anti-oxidant properties that might influence risk of mortality when consumed regularly [10–14]. Our results on boiled coffee may be related to the cholesterol rising effect of the diterpenes cafestol and kahweol [36]. In filtered coffee, these substances are left in the filter paper, sparing filtered coffee-drinkers of their potential harmful CVD-related effects. In our study, the association of total and filtered coffee with reduced all-cause and CVD mortality, is at large not evident in the boiled coffee group. Still, increased all-cause, CVD, and cancer mortality in the boiled coffee group is only found in the full cohort and not in the non-smokers, again pointing to the impact of smoking. Unlike their serum lipid raising effect, the coffee diterpenes may have favorable impact on cancer risk, due to their beneficial effect on oxidative stress and inflammatory processes [35]. [36]. As summarized by Ren et al., the coffee diterpenes may have an inhibitory effect at multiple stages of cancer development, including tumorigenesis, tumor cell proliferation, and metastasis [36]. The mechanisms involve inhibition of detoxifying CYP enzymes, induction of apoptosis, and inhibition of angiogenesis [36]. However, no such beneficial effect is evident in our data, not even in the never smokers.

Trigonelline was reported to have hypoglycemic, neuroprotective, anti-carcinogenic and antibacterial effects [34]. Coffee melanoidins are best known for their potential probiotic effect, but they also have a possible anti-carcinogenic effect [34]. Caffeine has a known central nervous system stimulatory effect, as well as an impact on cardiovascular health due to possible acute increase in heart rate and blood pressure [34]. Habitual coffee drinkers will experience less of this blood pressure rising effect due to increased tolerance of caffeine [37]. A recent meta-analysis that looked at both caffeinated and decaffeinated coffee consumption and all-cause mortality found no difference in the risk reduction (per 1 cup/day increments) from all-cause mortality between caffeinated and decaffeinated coffee [20]. This indicates that the effect of coffee on all-cause mortality might be unrelated to the caffeine consumption. Finally, chlorogenic acid, one of the major phenols present in coffee, may influence oxidative processes, although this may vary according to the roast of the coffee consumed [35].

Taken together, several substances from coffee can have a biological impact, and may influence all-cause, CVD, and cancer mortality. However, the potential bioactive concentration of substances, the underlying biological mechanisms, and potential synergistic effects from these substances in coffee are still not completely understood.

### Strengths and limitations

The main strengths of the present study are its prospective design and the relatively large sample size, with sufficient number of cases to detect differences between the coffee consumption groups in each of the outcomes. The response rate in the NOWAC study (52.7%) is similar to other population-based cohorts. A validation study showed that the study responders did not differ from the source population and negligible differences were found only in level of education [38]. Linkage to the Norwegian Central Population Register and the National Registry for Causes of Death via the unique person number allowed us to obtain virtually complete follow-up. Finally, a 24-h dietary recall study aiming to validate the FFQs used in the NOWAC cohort showed a high validity of information on coffee consumption (Spearman’s correlation coefficient r = 0.82) [39].

There are numerous limitations in our study. We did not have information on consumption of espresso, cappuccino, caffè latte, macchiato, or decaffeinated coffee for the entire studied cohort. However, the consumption of these types of coffee was relatively rare among Norwegian women at the time the baseline information were collected [40, 41]. In addition, information on condiments added in coffee, such as sugar, sweeteners, creamer, and milk were also available from a limited number of women in the cohort.

As coffee consumption was self-reported, misclassification cannot be ruled out, despite the results from the validation study. This potential misclassification was most likely non-differential among the comparison groups, hence, our observed associations may be underestimated.

We lacked sufficient details on smoking exposure (e.g. lifetime exposure to secondhand smoke) which would have allowed us to optimally adjust for smoking. As heavy coffee consumption is strongly associated with heavy smoking, residual confounding was highly probable in our study. Hence, the results from the analyses of the entire studied sample should be interpreted with caution. In order to remove the confounding effect of smoking, we repeated all the analyses in never smoker subgroup. However, the analyses of instant and boiled coffee consumption in never smokers were to a certain extent statistically underpowered, as the number of cases within the heavy consumption categories of these brewing types might have been too low to detect significant risk differences.

Finally, some residual confounding effect of the other adjustment covariates is probable, due to measurement error in self-reported life-style variables.

## Conclusion

Our data show that coffee consumption, in particular of the filtered brewing type, reduces the risk of cardiovascular deaths. The observed stronger association in never smokers indicates that a residual confounding by smoking is present, which likely explains the observed adverse association between coffee consumption and cancer mortality.

## Electronic supplementary material

Below is the link to the electronic supplementary material.

Supplementary material 1 (DOCX 23 kb)
