# Supplementary material for: Coffee consumption and overall and cause-specific mortality: the Norwegian Women and Cancer Study (NOWAC)
Source: Eur J Epidemiol. 2020 Jul 23;35(10):913–24. doi: 10.1007/s10654-020-00664-x (PMC7524812; doi:10.1007/s10654-020-00664-x)
Supplement: Supplementary file 1 — Supplementary material 1 (DOCX 23 kb) [file 10654_2020_664_MOESM1_ESM.docx]

**Supplementary table 1: Distribution of total, cardiovascular, and cancer deaths according to total, filtered, instant, and boiled coffee consumption at baseline, the Norwegian Women and Cancer Study, 1991-2016.**

|  | **Total deaths** | | | |
| --- | --- | --- | --- | --- |
|  | **Total coffee consumption**  **n (%)** | **Filtered coffee consumption**  **n (%)** | **Instant coffee consumption**  **n (%)** | **Boiled coffee consumption**  **n (%)** |
| **Light consumers**  **≤1 cup/day** | **2116 (17.1)** | **5628 (45.5)** | **11 300 (91.4)** | **9420 (76.2)** |
| **Low-moderate consumers**  **>1-4 cups/day** | **3672 (29.7)** | **2597 (21.0)** | **598 (4.8)** | **1102 (8.9)** |
| **High-moderate consumers**  **>4-6 cups/day** | **3615 (29.2)** | **2392 (19.4)** | **299 (2.4)** | **1025 (8.3)** |
| **Heavy consumers**  **>6 cups/day** | **2961 (24.0)** | **1747 (14.1)** | **167 (1.4)** | **817 (6.6)** |
|  | **Cardiovascular deaths** | | | |
|  | **Total coffee consumption**  **n (%)** | **Filtered coffee consumption**  **n (%)** | **Instant coffee consumption**  **n (%)** | **Boiled coffee consumption**  **n (%)** |
| **Light consumers**  **≤1 cup/day** | **347 (17.7)** | **952 (48.8)** | **1789 (91.6)** | **1454 (74.5)** |
| **Low-moderate consumers**  **>1-4 cups/day** | **609 (31.2)** | **390 (20-0)** | **94 (4.8)** | **190 (9.7)** |
| **High-moderate consumers**  **>4-6 cups/day** | **566 (29.0)** | **356 (18.2)** | **52 (2.7)** | **170 (8.7)** |
| **Heavy consumers**  **>6 cups/day** | **431 (22.1)** | **255 (13.1)** | **18 (0.9)** | **139 (7.1)** |
|  | **Cancer deaths** | | | |
|  | **Total coffee consumption**  **n (%)** | **Filtered coffee consumption**  **n (%)** | **Instant coffee consumption**  **n (%)** | **Boiled coffee consumption**  **n (%)** |
| **Light consumers**  **≤1 cup/day** | **884 (16.1)** | **2330 (42.5)** | **5031 (91.8)** | **4241 (77.4)** |
| **Low-moderate consumers**  **>1-4 cups/day** | **1612 (29.4)** | **1198 (21.9)** | **263 (4.8)** | **462 (8.4)** |
| **High-moderate consumers**  **>4-6 cups/day** | **1612 (29.4)** | **1118 (20.4)** | **111 (2.0)** | **444 (8.1)** |
| **Heavy consumers**  **>6 cups/day** | **1375 (25.1)** | **837 (15.3)** | **78 (1.4)** | **336 (6.1)** |

**Supplementary table 2: Hazard ratios (HRs) with 95% confidence intervals (CI) of all-cause mortality according to total, filtered, instant, and boiled coffee consumption in the Norwegian Women and Cancer Study.**

| **All-cause mortality** | | | | | | | | |
| --- | --- | --- | --- | --- | --- | --- | --- | --- |
| **Coffee consumption** | **Total coffee consumption** | | **Filtered coffee consumption** | | **Instant coffee consumption** | | **Boiled coffee consumption** | |
|  | **Multivariable^1^** | **Multivariable^2^** | **Multivariable^3^** | **Multivariable^4^** | **Multivariable^3^** | **Multivariable^4^** | **Multivariable^3^** | **Multivariable^4^** |
|  | HR  95% CI | HR  95% CI | HR  95% CI | HR  95% CI | HR  95% CI | HR  95% CI | HR  95% CI | HR  95% CI |
| **≤1 cup/day** | 1.00 | 1.00 | 1.00 | 1.00 | 1.00 | 1.00 | 1.00 | 1.00 |
| **>1-4 cups/day** | 0.89  (0.83-0.94) | 0.90  (0.84-0.95) | 0.91  (0.86-0.97) | 0.91  (0.85-0.96) | 0.92  (0.84-1.02) | 0.91  (0.83-1.00) | 1.02  (0.94-1.11) | 0.99  (0.91-1.07) |
| **>4--6 cups/day** | 0.98  (0.92-1.05) | 0.89  (0.83-0.94) | 1.01  (0.95-1.07) | 0.90  (0.85-0.96) | 1.12  (0.98-1.28) | 0.97  (0.85-1.11) | 1.10  (1.02-1.20) | 0.96  (0.99-1.05) |
| **>6 cups/day** | 1.34  (1.25-1.43) | 1.02  (0.95-1.09) | 1.45  (1.36-1.56) | 1.09  (1.01-1.17) | 1.42  (1.19-1.71) | 1.11  (0.93-1.34) | 1.31  (1.19-1.43) | 0.98  (0.89-1.08) |

^1^Adjusted for body mass index (cat.), alcohol consumption (g/day) (cat.), physical activity (cat.), years of attained education (cat.).

^2^Adjusted for smoking status, age at smoking initiation, number of pack-years smoked, body mass index (cat.), alcohol consumption (g/day) (cat.), physical activity (cat.), years of attained education (cat.).

^3^Adjusted for body mass index (cat.), alcohol consumption (g/day) (cat.), physical activity (cat.), years of attained education (cat.), and mutually adjusted for the consumption of coffee brewed with two other methods (cat.).

^4^Adjusted for smoking status, age at smoking initiation, number of pack-years smoked, body mass index (cat.), alcohol consumption (g/day) (cat.), physical activity (cat.), years of attained education (cat.), and mutually adjusted for the consumption of coffee brewed with two other methods (cat.).

Cat.: categorical;

**Supplementary table 3: Hazard ratios (HRs) with 95% confidence intervals (CI) of cardiovascular mortality according to total, filtered, instant, and boiled coffee consumption in the Norwegian Women and Cancer Study.**

| **Cardiovascular mortality** | | | | | | | | |
| --- | --- | --- | --- | --- | --- | --- | --- | --- |
| **Coffee consumption** | **Total coffee consumption** | | **Filtered coffee consumption** | | **Instant coffee consumption** | | **Boiled coffee consumption** | |
|  | **Multivariable^1^** | **Multivariable^2^** | **Multivariable^3^** | **Multivariable^4^** | **Multivariable^3^** | **Multivariable^4^** | **Multivariable^3^** | **Multivariable^4^** |
|  | HR  95% CI | HR  95% CI | HR  95% CI | HR  95% CI | HR  95% CI | HR  95% CI | HR  95% CI | HR  95% CI |
| **≤1 cup/day** | 1.00 | 1.00 | 1.00 | 1.00 | 1.00 | 1.00 | 1.00 | 1.00 |
| **>1-4 cups/day** | 0.84  (0.71-0.98) | 0.85  (0.72-1.00) | 0.81  (0.69-0.95) | 0.80  (0.68-0.94) | 0.96  (0.75-1.22) | 0.94  (0.74-1.20) | 1.00  (0.81-1.22) | 0.93  (0.76-1.15) |
| **>4-6 cups/day** | 0.92  (0.78-1.08) | 0.79  (0.67-0.94) | 0.93  (0.79-1.10) | 0.80  (0.67-0.94) | 1.14  (0.82-1.58) | 0.95  (0.68-1.32) | 1.07  (0.87-1.32) | 0.89  (0.72-1.10) |
| **>6 cups/day** | 1.23  (1.03-1.46) | 0.85  (0.71-1.02) | 1.35  (1.12-1.62) | 0.92  (0.76-1.12) | 0.99  (0.58-1.69) | 0.74  (0.43-1.27) | 1.37  (1.08-1.73) | 0.90  (0.70-1.15) |

^1^Adjusted for body mass index (cat.), alcohol consumption (g/day) (cat.), physical activity (cat.), years of attained education (cat.).

^2^Adjusted for smoking status, age at smoking initiation, number of pack-years smoked, body mass index (cat.), alcohol consumption (g/day) (cat.), physical activity (cat.), years of attained education (cat.).

^3^Adjusted for body mass index (cat.), alcohol consumption (g/day) (cat.), physical activity (cat.), years of attained education (cat.), and mutually adjusted for the consumption of coffee brewed with two other methods (cat.).

^4^Adjusted for smoking status, age at smoking initiation, number of pack-years smoked, body mass index (cat.), alcohol consumption (g/day) (cat.), physical activity (cat.), years of attained education (cat.), and mutually adjusted for the consumption of coffee brewed with two other methods (cat.).

Cat.: categorical;

**Supplementary table 4: Hazard ratios (HRs) with 95% confidence intervals (CI) of cancer mortality according to total, filtered, instant, and boiled coffee consumption in the Norwegian Women and Cancer Study.**

| **Cancer mortality** | | | | | | | | |
| --- | --- | --- | --- | --- | --- | --- | --- | --- |
| **Coffee consumption** | **Total coffee consumption** | | **Filtered coffee consumption** | | **Instant coffee consumption** | | **Boiled coffee consumption** | |
|  | **Multivariable^1^** | **Multivariable^2^** | **Multivariable^3^** | **Multivariable^4^** | **Multivariable^3^** | **Multivariable^4^** | **Multivariable^3^** | **Multivariable^4^** |
|  | HR  95% CI | HR  95% CI | HR  95% CI | HR  95% CI | HR  95% CI | HR  95% CI | HR  95% CI | HR  95% CI |
| **≤1 cup/day** | 1.00 | 1.00 | 1.00 | 1.00 | 1.00 | 1.00 | 1.00 | 1.00 |
| **>1-4 cups/day** | 0.94  (0.86-1.03) | 0.95  (0.86-1.04) | 0.97  (0.89-1.06) | 0.97  (0.89-1.05) | 0.98  (0.85-1.12) | 0.96  (0.83-1.10) | 1.03  (0.92-1.16) | 1.00  (0.89-1.13) |
| **>4-6 cups/day** | 1.02  (0.93-1.12) | 0.94  (0.86-1.04) | 1.05  (0.96-1.15) | 0.97  (0.88-1.06) | 0.95  (0.77-1.18) | 0.87  (0.70-1.08) | 1.18  (1.05-1.33) | 1.07  (0.94-1.20) |
| **>6 cups/day** | 1.40  (1.27-1.54) | 1.14  (1.03-1.26) | 1.53  (1.39-1.69) | 1.23  (1.11-1.36) | 1.73  (1.35-2.21) | 1.40  (1.09-1.81) | 1.21  (1.06-1.40) | 0.98  (0.85-1.13) |

^1^Adjusted for body mass index (cat.), alcohol consumption (g/day) (cat.), physical activity (cat.), years of attained education (cat.).

^2^Adjusted for smoking status, age at smoking initiation, number of pack-years smoked, body mass index (cat.), alcohol consumption (g/day) (cat.), physical activity (cat.), years of attained education (cat.).

^3^Adjusted for body mass index (cat.), alcohol consumption (g/day) (cat.), physical activity (cat.), years of attained education (cat.), and mutually adjusted for the consumption of coffee brewed with two other methods (cat.).

^4^Adjusted for smoking status, age at smoking initiation, number of pack-years smoked, body mass index (cat.), alcohol consumption (g/day) (cat.), physical activity (cat.), years of attained education (cat.), and mutually adjusted for the consumption of coffee brewed with two other methods (cat.).

Cat.: categorical;
